# Elderly woman with purple rash on eyelids

**DOI:** 10.11604/pamj.2018.30.80.15037

**Published:** 2018-05-29

**Authors:** Fred Bernardes Filho, Breno Nery

**Affiliations:** 1 Dermatology Division, Department of Medical Clinics, Ribeirão Preto Medical School, University of São Paulo, Ribeirão Preto, Brazil; 2 Emergency Department, Hospital Imaculada Conceição da Sociedade Portuguesa de Beneficência, Ribeirão Preto, São Paulo, Brazil; 3 Department of Neurosurgery, Hospital Imaculada Conceição da Sociedade Portuguesa de Beneficência, Ribeirão Preto, São Paulo, Brazil

**Keywords:** Dermatomyositis, connective tissue disease, creatine kinase

## Image in medicine

A 62-year-old woman presented with purple rash on her eyelids, erythematous and violaceous rash over the lateral thighs, and proximal muscle weakness. On physical examination, she had a pulse rate of 90/min and blood pressure of 138/90 mmHg. Dermatological examination showed reddish-purple rash on the upper eyelids and violaceous erythema on the lateral surface of the thighs. Laboratory testing was remarkable for elevated creatine kinase at 4,460. White blood cells, liver enzymes, electrolytes, antinuclear antibodies, cranial, abdominal and thorax tomography were unremarkable. Serology tests for hepatitis B, and C, HIV, syphilis, spotted fever and Lyme borreliosis were negative. The diagnosis of dermatomyositis was performed and the treatment was started with prednisone 1mg/kg daily. Dermatomyositis is a connective tissue disease that combines myopathy and distinctive skin manifestations. Creatine kinase is the muscle enzyme most widely used to diagnose polymyositis/dermatomyositis and to follow therapeutic response. There are hallmark cutaneous signs described for dermatomyositis that may or may not parallel the inflammatory myopathy. These include: violaceous to dusky erythematous rash with or without edema on periorbital skin (heliotrope rash), scaly erythematous eruption on the dorsum of hands, metacarpophalangeal joints, proximal interphalangeal joints, knees and elbows (Gottron's sign), confluent macular violaceous erythema on the anterior neck and chest ("V" sign) and confluent macular violaceous erythema present on the lateral side of hip and thighs (Holster sign). The cutaneous disease in dermatomyositis is photodistributed and often photoaggravated. The importance of recognizing dermatological signs in dermatomyositis is due to the cutaneous lesions precede muscle disease in one third to one half of patients.

**Figure 1:**
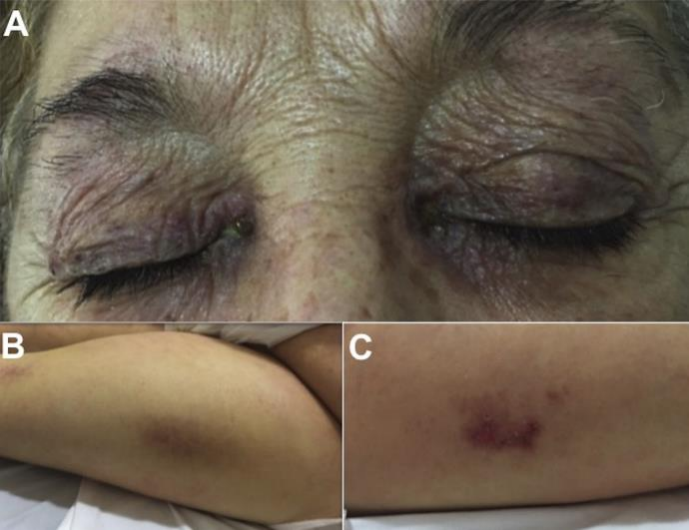
A) a reddish-purple rash on the upper eyelids (Heliotrope rash); B) violaceous erythema on the lateral surface of the left thigh (Holster sign); C) confluent macular violaceous erythema on the lateral side of right thigh (Holster sign)

